# Supplementary material for: Astrocytes regulate brain extracellular pH via a neuronal activity-dependent bicarbonate shuttle
Source: Nat Commun. 2020 Oct 8;11:5073. doi: 10.1038/s41467-020-18756-3 (PMC7545092; doi:10.1038/s41467-020-18756-3)
Supplement: Supplementary file 1 — Supplementary information [file 41467_2020_18756_MOESM1_ESM.pdf]

Supplementary Information

Astrocytes regulate brain extracellular pH via a neuronal activity-dependent bicarbonate shuttle

Theparambil, Hosford et al.

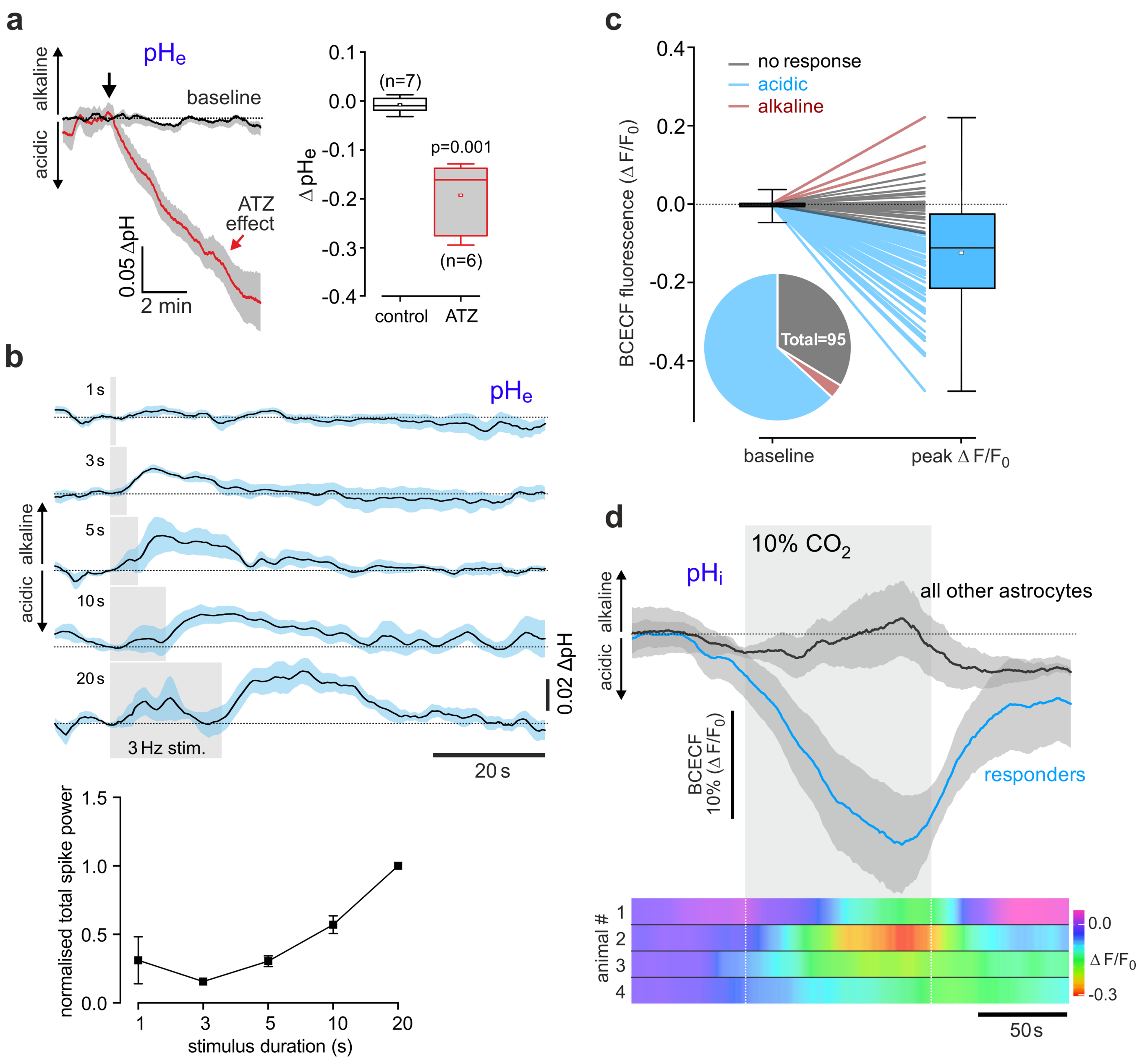

Supplementary Figure 1| pH regulation in the somatosensory cortex. **a,** The effect of acetazolamide (ATZ) on extracellular pH (pH_e) in the somatosensory cortex of anesthetized rats. Administration of ATZ (10 mg kg^{-1}, iv) leads to a progressive decrease in brain pH_e as evident from a decrease in pH-sensitive electrochemical current recorded by fast cyclic voltammetry. Traces illustrate averaged (means ± SEM) changes in pH-sensitive current. Box-and-whisker plot illustrates peak changes in pH_e in response to ATZ. *p*-value, Mann Whitney-U test. **b**, Time course of pH_e changes in the right forelimb region of the somatosensory (S1FL) cortex of anaesthetised mice induced by electrical stimulation (3 Hz, 1.5 mA) of the contralateral forepaw with durations of 1, 3, 5, 10 and 20 s, applied in random order. Traces illustrate averaged (means ± SEM) changes in pH-sensitive electrochemical current evoked by somatosensory stimulation recorded in 7 animals. Group data (means ± SEM) illustrate normalised total spike power recorded during the stimulations. **c,** Peak changes in BCECF fluorescence in individual S1FL astrocytes (n=95; recorded in 4 mice) induced by 10% inspired CO_2. **d,** Time course of intracellular pH (pH_i) changes recorded in S1FL astrocytes in response to 10% inspired CO_2. Traces illustrate averaged (means ± SEM) changes in BCECF fluorescence recorded in cortical astrocytes that showed peak change in $\Delta F/F_0 \geq 2$ SD of baseline fluorescence (responders; 63% of the whole population) and all other astrocytes. False colour plots illustrate averaged changes in BCECF fluorescence in all SR101-labelled cells in each individual animal. In box-and-whisker plots the central dot indicates the mean, the central line indicates the median, the box limits indicate the upper and lower quartiles and the whiskers show the minimum-maximum range of the data.

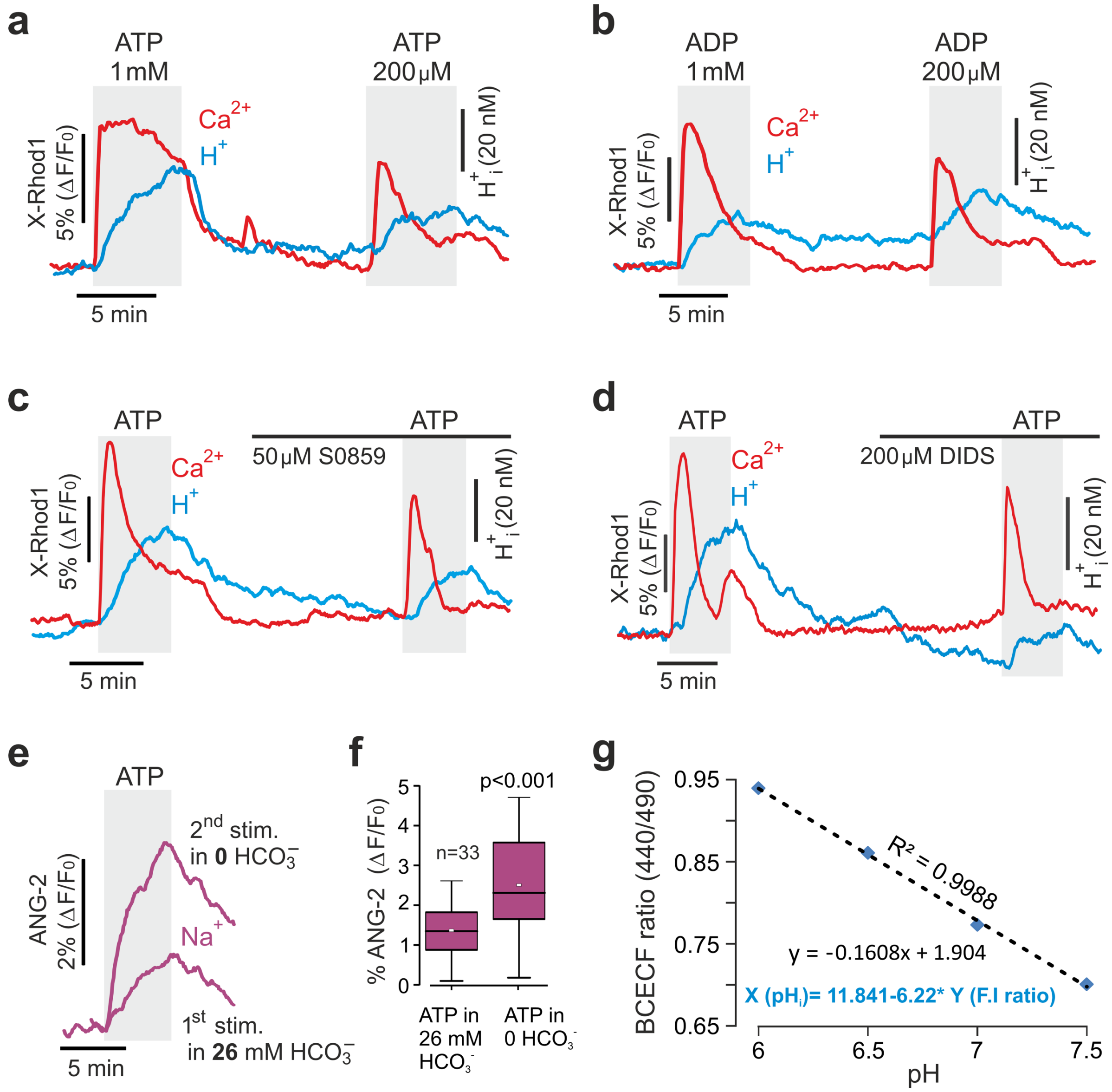

Supplementary Figure 2| Purinergic control of astrocyte bicarbonate release. **a-b,** Representative examples illustrating ATP- and ADP-induced intracellular [H^+] and [Ca^{2+}] responses recorded in 18 astrocytes in culture. **c-d**, Representative examples showing the effect of sodium-bicarbonate transporter inhibitors S0859 (50 μM) and DIDS (200 μM) on ATP-evoked intracellular [H^+] and [Ca^{2+}] responses in 14 astrocytes in culture. **e,** Representative example illustrating the effect of ATP on intracellular [Na^+] in the presence and absence of bicarbonate in the buffer. The averaged traces of changes in [Na^+]$_i$ in 11 astrocytes are shown. **f,** Summary data illustrating peak [Na^+]$_i$ increases induced by ATP in the presence and absence of extracellular HCO_3^-. In the box-and-whisker plot the central dot indicates the mean, the central line indicates the median, the box limits indicate the upper and lower quartiles and the whiskers extend to 1.5 IQR from the quartiles. *p*-value, one-Way ANOVA. **g,** Four-point calibration of BCECF-fluorescence obtained using Nigericin (10 μM)/high K^+ (130 mM) method. A linear fit was constructed; the equation given was used to convert BCECF fluorescence values to intracellular pH, and then to intracellular [H^+].

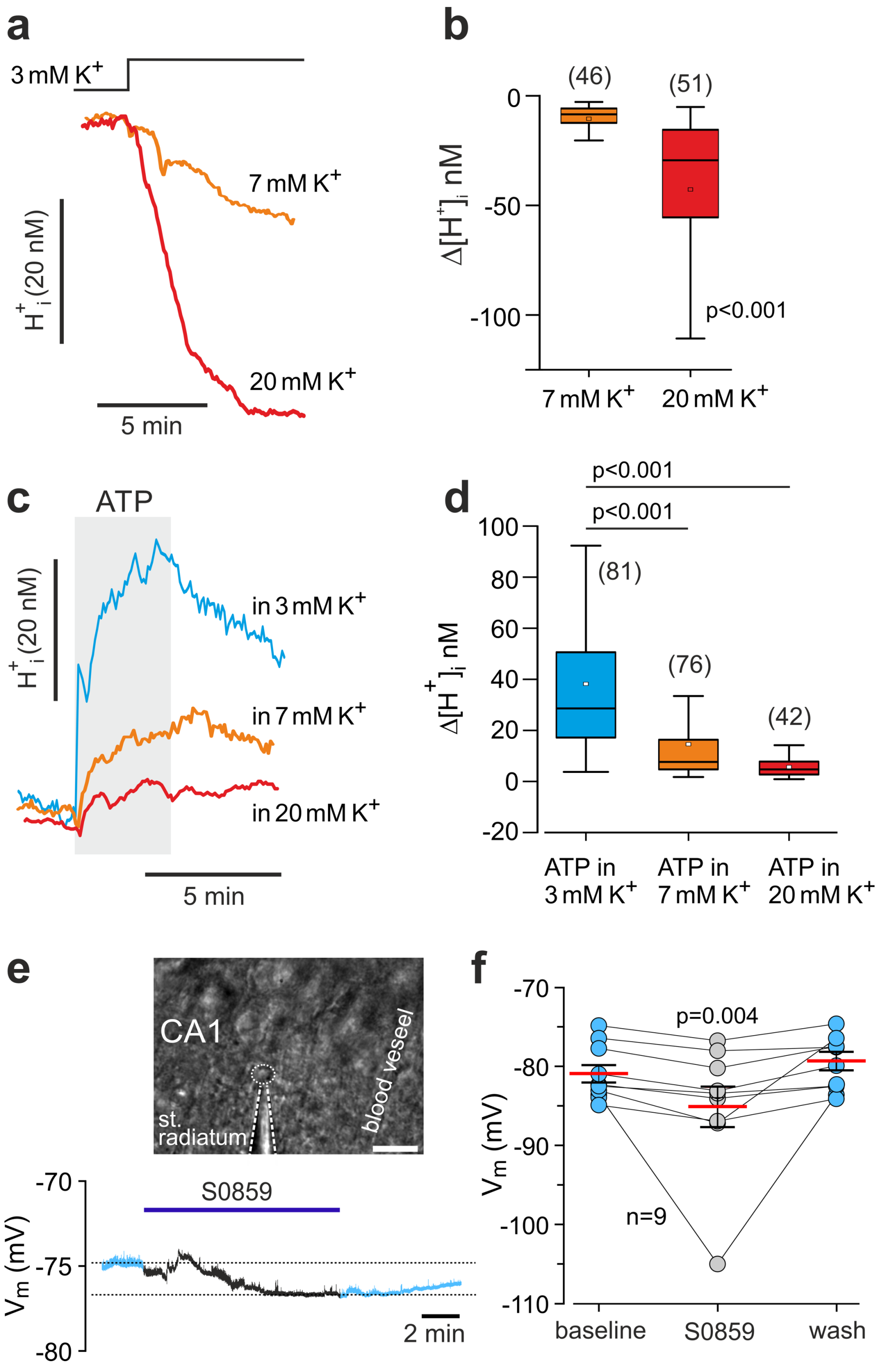

Supplementary Figure 3| NBCe1-mediated bicarbonate transport in astrocytes is governed by the membrane potential. **a**, Representative traces illustrating the effect of raising extracellular [K$^+$] on intracellular [H$^+$] recorded in 14 astrocytes in culture. **b**, Summary data illustrating the magnitude of $[H^+]_i$ decreases in astrocytes in response to membrane depolarization induced by 7 and 20 mM [K$^+$]. **c**, Representative traces showing the effect of high extracellular [K$^+$] on ATP (1 mM)-induced intracellular [H$^+$] responses recorded in 15 astrocytes in culture. **d**, Summary data illustrating peak increases in $[H^+]_i$ in response to ATP at different extracellular K$^+$ concentrations. In the box-and-whisker plots the central dot indicates the mean, the central line indicates the median, the box limits indicate the upper and lower quartiles and the whiskers extend to 1.5 IQR from the quartiles. *p*-values, one-Way ANOVA. **e**, Representative example of whole-cell recording from an astrocyte in an acute hippocampal slice, illustrating changes in the resting membrane potential (V_m) in response to NBC blockade with S0859 (50 μM). Image illustrates the position of the recorded astrocyte relative to the CA1 neuronal layer. Scale bar = 20 μm. **f**, Summary data (individual values and means ± SEM) illustrating the effect of S0859 on membrane potential in hippocampal astrocytes (9 individual cells/slices, obtained from 4 animals). *p*-value, Wilcoxon signed rank test.

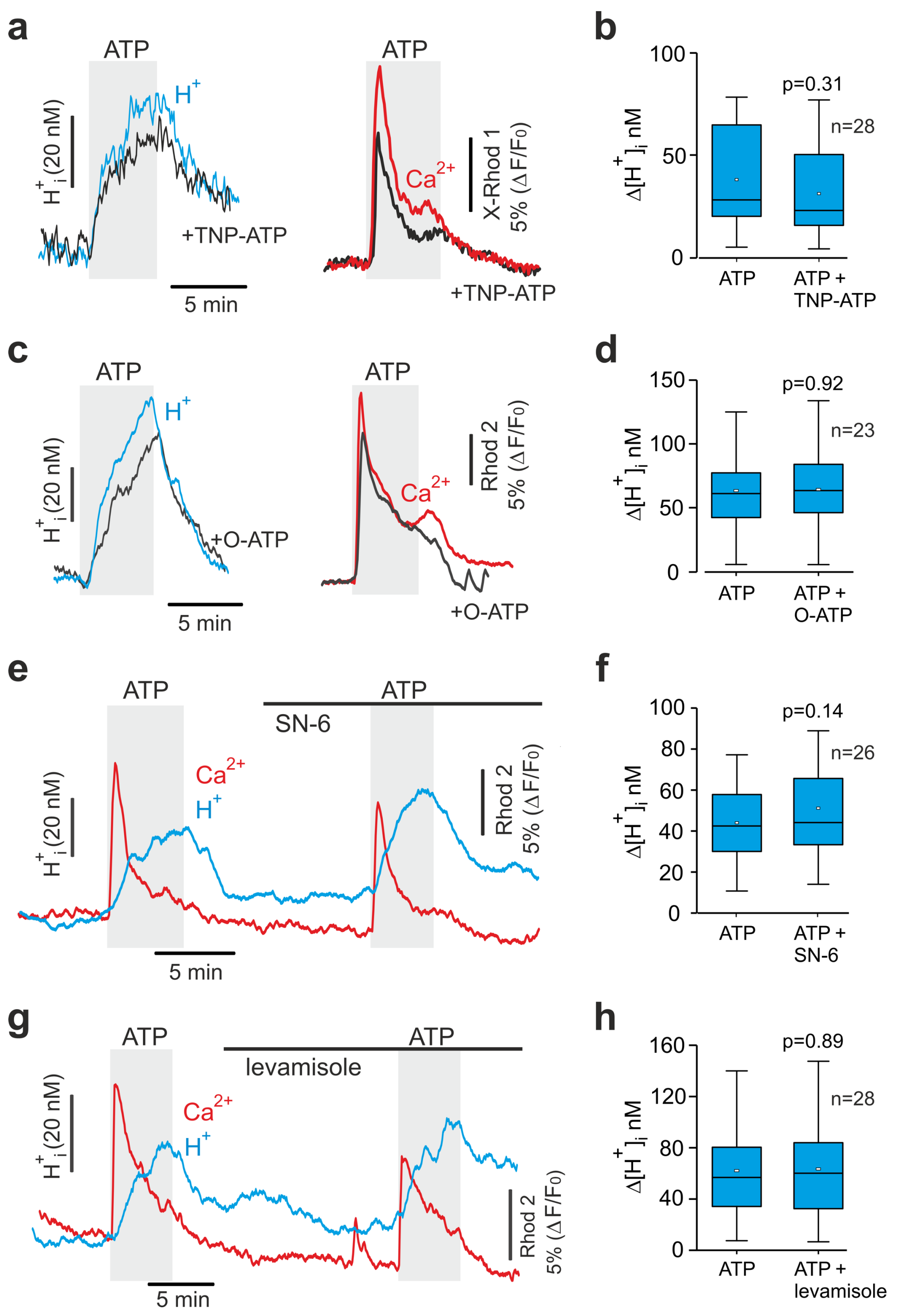

Supplementary Figure 4| The effect of pharmacological blockade of P2X receptors, sodium/calcium exchange and alkaline phosphatase on ATP-induced $[H^+]_i$ responses in astrocytes. **a,** Representative recordings showing the effect of P2X receptor antagonist TNP-ATP (10 μM) on ATP-induced $[H^+]_i$ and $[Ca^{2+}]_i$ responses in cultured astrocytes. Averaged traces of $[H^+]_i$ and $[Ca^{2+}]_i$ changes recorded in 12 astrocytes are shown. **b,** Summary data illustrating peak increases in $[H^+]_i$ in response to ATP in the absence and presence of TNP-ATP. **c,** Representative recordings showing the effect of $P2X_7$ receptor antagonist O-ATP (100 μM) on ATP-induced $[H^+]_i$ and $[Ca^{2+}]_i$ responses in astrocytes (n=11). **d,** Summary data illustrating peak increases in $[H^+]_i$ in response to ATP in the absence and presence of O-ATP. **e,** Representative recordings showing the effect of the sodium/calcium exchanger inhibitor SN-6 (20 μM) on ATP-induced $[H^+]_i$ and $[Ca^{2+}]_i$ responses in astrocytes (n=12). **f,** Summary data illustrating peak increases in $[H^+]_i$ in response to ATP in the absence and presence of SN-6. **g,** Representative recordings showing the effect of alkaline phosphatase inhibitor levamisole (500 μM) on ATP-induced $[H^+]_i$ and $[Ca^{2+}]_i$ responses in astrocytes (n=11). **h,** Summary data illustrating peak increases in $[H^+]_i$ in response to ATP in the absence and presence of levamisole. In the box-and-whisker plots the central dot indicates the mean, the central line indicates the median, the box limits indicate the upper and lower quartiles and the whiskers extend to 1.5 IQR from the quartiles. *p*-values, one-Way ANOVA.

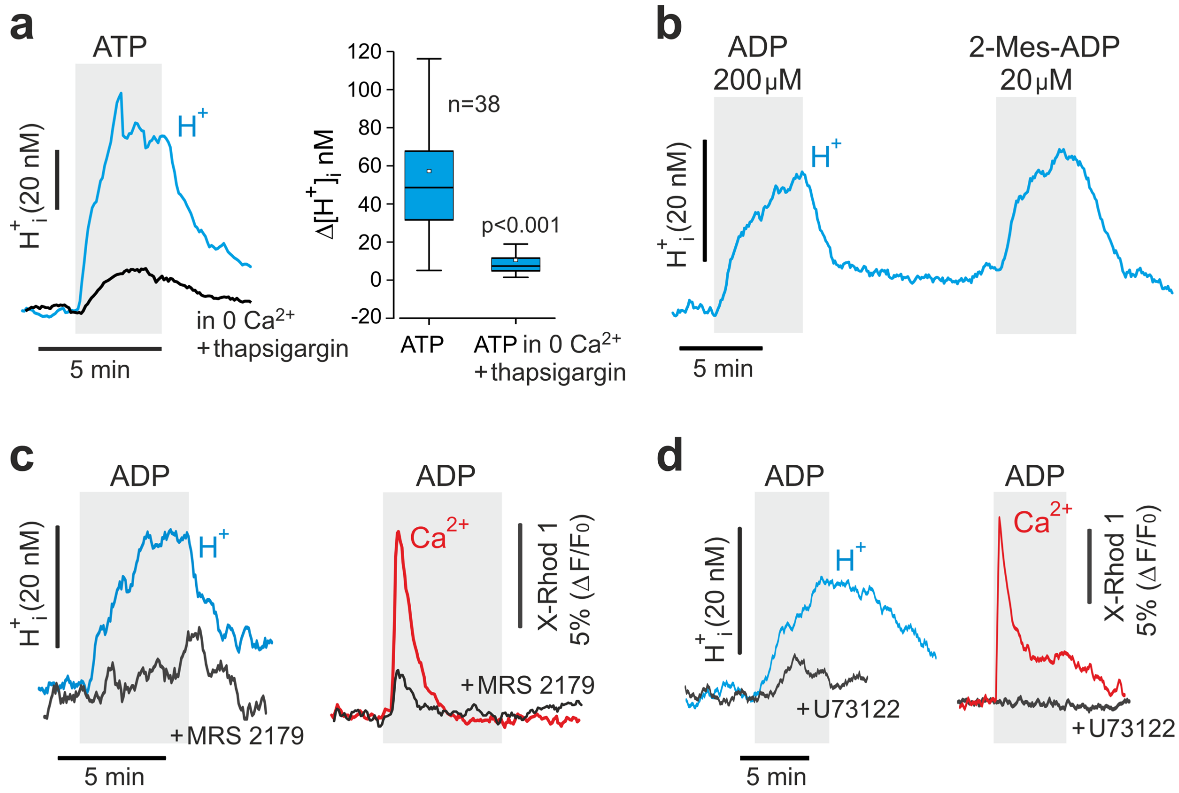

Supplementary Figure 5| The role of $P2Y_1$ receptors. **a**, ATP-induced intracellular acidification in astrocytes is inhibited after depletion of intracellular Ca^{2+} stores with thapsigargin (1 μM) in the absence of extracellular Ca^{2+}. Averaged traces of ATP-induced $[H^+]_i$ responses in 15 astrocytes are shown. Summary data illustrate the effects of extracellular Ca^{2+} removal combined with depletion of intracellular Ca^{2+} stores with thapsigargin (1 μM) on peak increases in $[H^+]_i$ induced by ATP (1 mM) in astrocytes. In the box-and-whisker plot the central dot indicates the mean, the central line indicates the median, the box limits indicate the upper and lower quartiles and the whiskers extend to 1.5 IQR from the quartiles. *p*-value, one-Way ANOVA. **b,** Representative recording illustrating the effects of ADP and $P2Y_1$ receptor agonist 2-Mes-ADP on $[H^+]_i$ in astrocytes. Averaged traces of $[H^+]_i$ changes recorded in 13 astrocytes in culture are shown. **c,** Representative recordings showing the effect of $P2Y_1$ receptor antagonist MRS2179 (20 μM) on ADP-induced $[H^+]_i$ and $[Ca^{2+}]_i$ responses in astrocytes (n=12). **d,** Representative recordings showing the effect of phospholipase C inhibitor U73122 (10 μM) on ADP-induced $[H^+]_i$ and $[Ca^{2+}]_i$ responses in astrocytes. Averaged traces of $[H^+]_i$ and $[Ca^{2+}]_i$ responses in 11 astrocytes are shown.
