# Supplementary material for: Astrocytes regulate brain extracellular pH via a neuronal activity-dependent bicarbonate shuttle
Source: Nat Commun. 2020 Oct 8;11:5073. doi: 10.1038/s41467-020-18756-3 (PMC7545092; doi:10.1038/s41467-020-18756-3)
Supplement: Supplementary file 3 — Reporting Summary [file 41467_2020_18756_MOESM3_ESM.pdf]

natureresearch

Corresponding author(s): Alexander V. Gourine

Last updated by author(s): 01/09/2020

Reporting Summary

Nature Research wishes to improve the reproducibility of the work that we publish. This form provides structure for consistency and transparency in reporting. For further information on Nature Research policies, see Authors & Referees and the Editorial Policy Checklist .

Statistics

For all statistical analyses, confirm that the following items are present in the figure legend, table legend, main text, or Methods section.

n/a	Confirmed	
☐	☒	The exact sample size (*n*) for each experimental group/condition, given as a discrete number and unit of measurement
☐	☒	A statement on whether measurements were taken from distinct samples or whether the same sample was measured repeatedly
☐	☒	The statistical test(s) used AND whether they are one- or two-sided<br>*Only common tests should be described solely by name; describe more complex techniques in the Methods section.*
☒	☐	A description of all covariates tested
☐	☒	A description of any assumptions or corrections, such as tests of normality and adjustment for multiple comparisons
☐	☒	A full description of the statistical parameters including central tendency (e.g. means) or other basic estimates (e.g. regression coefficient) AND variation (e.g. standard deviation) or associated estimates of uncertainty (e.g. confidence intervals)
☐	☒	For null hypothesis testing, the test statistic (e.g. *F*, *t*, *r*) with confidence intervals, effect sizes, degrees of freedom and *P* value noted<br>*Give P values as exact values whenever suitable.*
☒	☐	For Bayesian analysis, information on the choice of priors and Markov chain Monte Carlo settings
☒	☐	For hierarchical and complex designs, identification of the appropriate level for tests and full reporting of outcomes
☒	☐	Estimates of effect sizes (e.g. Cohen's *d*, Pearson's *r*), indicating how they were calculated

Our web collection on statistics for biologists contains articles on many of the points above.

Software and code

Policy information about availability of computer code

Data collection	Power 1401, Spike2-Version7, Axon-pClamp-version 10.2, IQ3 imaging-Version 6.3, Olympus FluoView-Version 4
Data analysis	Spike2-Version7; GraphPad-Prism-Version8; Axon-pClamp version 10.2; Image J-Version 1.52P, Seurat package73 (v.3.1.4) in R-Studio (v.3.6.0, "Planting of a Tree")

For manuscripts utilizing custom algorithms or software that are central to the research but not yet described in published literature, software must be made available to editors/reviewers. We strongly encourage code deposition in a community repository (e.g. GitHub). See the Nature Research guidelines for submitting code & software for further information.

Data

Policy information about availability of data

All manuscripts must include a data availability statement. This statement should provide the following information, where applicable:

- Accession codes, unique identifiers, or web links for publicly available datasets
- A list of figures that have associated raw data
- A description of any restrictions on data availability

The data that support the findings in this study are included within the supplementary material and available from the corresponding author upon request. The source data underlying Figs 1a,b,d,e, 2d, 3f,h, 5c,e, 6b,g,h, and 7e,f,g and Supplementary Figs 1a-d, 2f,g, 3b,d,f, 4b,d,f,h and 5a are provided as a Source Data file. Single cell RNA-sequencing (RNAseq) source data underlying Fig 4 are available from a publicly available database (http://mousebrain.org/).

Field-specific reporting

Please select the one below that is the best fit for your research. If you are not sure, read the appropriate sections before making your selection.

Life sciences study design

All studies must disclose on these points even when the disclosure is negative.

Sample size	The paper describes the results of the experiments performed using in vitro (primary culture and acute slices) and in vivo animal preparations. Power calculations for the in vivo animal studies are described below. The significance was set at 0.05 and the beta was set at 0.20. From years of relevant research experience, we expect to detect a significant difference with a minimum of 5 animals per experimental group, if the treatments cause differences between means that are as large as 2.25 standard deviations (SD), likely to be a physiologically significant difference. If the difference is as small as 1.75 SD, we increase sample sizes to 9. Although, increasing the sample sizes per group to 15 may enable to detect a difference between means of 1.25 SD, it is doubtful these differences would have a biological significance. For the analysis of the in vitro data, number of cells from at least 3 or more experimental animals were tested to assess the statistical differences between the experimental groups.
Data exclusions	No data was excluded from the analysis
Replication	All experiments were independently conducted at least three times in the in vitro studies, and at least 5 times in the in vivo studies. All attempts to replicate the findings were consistently successful in the present study.
Randomization	Randomization is not relevant to this study, since experimental subjects (C57Bl/6J mice and Sprague-Dawley rats) were obtained from a genetically homogeneous colony and then assigned to different experimental groups according to the treatment.
Blinding	All experimenters were blinded to the condition or treatment of experimental subjects and to the identity of the experimental groups.

Reporting for specific materials, systems and methods

We require information from authors about some types of materials, experimental systems and methods used in many studies. Here, indicate whether each material, system or method listed is relevant to your study. If you are not sure if a list item applies to your research, read the appropriate section before selecting a response.

Materials & experimental systems

n/a	Involved in the study	
☐	☒	Antibodies
☒	☐	Eukaryotic cell lines
☒	☐	Palaeontology
☐	☒	Animals and other organisms
☒	☐	Human research participants
☒	☐	Clinical data

Methods

n/a	Involved in the study	
☒	☐	ChIP-seq
☒	☐	Flow cytometry
☒	☐	MRI-based neuroimaging

Antibodies

Antibodies used	Primary mouse monoclonal anti-NBCe1 (Santa Cruz Biotechnology, Cat # sc-515543, Lot # I2216); mouse polyclonal anti-MAP2 antibodies (Sigma, Cat # M-1406), secondary anti-mouse antibody Dylight 488 (ThermoFisher, Cat #35502).
Validation	The antibodies were validated using negative control protocols and using cell/tissue samples of specific gene knockout animals. We employ these antibodies routinely and we have published research articles demonstrating their specificity. In the present study immunohistochemistry was used to show that tamoxifen- induced recombination is taking place specifically in astrocytes of NBCe1 flox/flox:GLAST CreERT2/+ mice. The specificity of anti-NBCe1 antibody was confirmed by observing lack of immunostaining in cultured astrocytes of global NBCe1 knockout mice. The validation data are available from the corresponding author upon request.

Animals and other organisms

Policy information about studies involving animals; ARRIVE guidelines recommended for reporting animal research

Laboratory animals	Young Sprague-Daweley rats(P21-25) and pups (p2-3) of both sexes, young adult C57Bl/6J mice (3-4 mo old) and pups (p2-3) of both sexes.
Wild animals	The study did not involve any wild animals
Field-collected samples	The study did not involve any field collected samples
Ethics oversight	All animal experimentations were performed in accordance with the European Commission Directive 2010/63/EU (European Convention for the Protection of Vertebrate Animals used for Experimental and Other Scientific Purposes) and the UK Home Office (Scientific Procedures) Act (1986) with project approval from the Institutional Animal Care and Use Committees of the

University College London and Centro de Estudios Científicos.

Note that full information on the approval of the study protocol must also be provided in the manuscript.
